# Co-micronized Palmitoylethanolamide/Polydatin Treatment Causes Endometriotic Lesion Regression in a Rodent Model of Surgically Induced Endometriosis

**DOI:** 10.3389/fphar.2016.00382

**Published:** 2016-10-14

**Authors:** Rosanna Di Paola, Roberta Fusco, Enrico Gugliandolo, Rosalia Crupi, Maurizio Evangelista, Roberta Granese, Salvatore Cuzzocrea

**Affiliations:** ^1^Department of Chemical, Biological, Pharmaceutical and Environmental Sciences, University of MessinaMessina, Italy; ^2^Institute of Anaesthesiology and Reanimation, Catholic University of the Sacred HeartRome, Italy; ^3^Department of Human Pathology, University of MessinaMessina, Italy; ^4^Department of Pharmacological and Physiological Science, Saint Louis University, Saint LouisMO, USA

**Keywords:** endometriosis, palmitoylethanolamide, polydatin, rat, treatment

## Abstract

Endometriosis is a chronic, painful disease characterized by the presence of endometrial glands and stroma outside the uterine cavity. Palmitoylethanolamide (PEA), an endogenous fatty acid amide, has anti-inflammatory and neuroprotective effects. PEA lacks free radical scavenging activity, unlike polydatin (PLD), a natural precursor of resveratrol. The aim of this study was to investigate the effect of orally administered co-micronized PEA/polydatin [m(PEA/PLD)] in an autologous rat model of surgically induced endometriosis. Endometriosis was induced in female Wistar albino rats by auto-transplantation of uterine squares (implants) into the intestinal mesentery and peritoneal cavity. Rats were distributed into one control group and one treatment group (10 animals each): m(PEA/PLD) 10 mg/kg/day. At 28 days after surgery the relative volume of the endometrioma was determined. Endometrial-like tissue was confirmed by histology: Masson trichrome and toluidine blue were used to detect fibrosis and mast cells, respectively. The treated group displayed a smaller cyst diameter, with improved fibrosis score and mast cell number decrease. m(PEA/PLD) administration decreased angiogenesis (vascular endothelial growth factor), nerve growth factor, intercellular adhesion molecule, matrix metalloproteinase 9 expression, and lymphocyte accumulation. m(PEA/PLD) treatment also reduced peroxynitrite formation, (poly-ADP)ribose polymerase activation, IkBα phosphorylation and nuclear facor-kB traslocation in the nucleus. Our results suggested that m(PEA/PLD) may be of use to inhibit development of endometriotic lesions in rats.

## Introduction

Endometriosis is a painful chronic disease characterized by the presence of endometrial glands and stroma outside the uterine cavity, predominantly, in the pelvic compartment. It affects ∼5% (Vigano et al., 2004) of women in reproductive age and it is associated with pelvic pain and infertility (Giudice, 2010). It has a multifactorial etiology including hormonal, genetic, and immunological aspects. The exact mechanism of the pathology is still undefined; The most widely accepted theory, the retrograde menstruation theory, was proposed by Sampson (1927). It postulates that epithelial cells and endometrial stroma regurgitate outside the fallopian tubes during menstruation. Subsequently, they will implant and grow into peritoneum and ovary due to a pressure gradient probably from dyssynergic uterine contraction (Groothuis et al., 2005). Other theories on the endometriosis pathogenesis include the endometrial steam cell implantation (an expansion of the Sampson theory), immune dysfunction and Mullerian remnant abnormalities (Burney and Giudice, 2012). The disease has a genetic etiology, with a hereditability of ∼51% (Nyholt et al., 2012). Seven risk loci manifested a robust association with endometriosis (Pagliardini et al., 2013). There is a significant overlap in polygenic risk for endometriosis between the European and Japanese GWA cohorts (Nyholt et al., 2012). In addition, endometriosis is considered an estrogen-dependent disorder. An alteration of estrogens signaling are present in the ectopic tissue of patients affected by disease; in particular elevated expression of Extrogen receptor β is predicted to play a role in the development of endometriosis; probably, it contributes to the resistance to progesterone action in some of patients suffering from endometriosis (Bulun et al., 2012).

American Society of Reproductive Medicine report that “Unfortunately, there is no permanent cure for this condition, so it often requires a life-long management plan with the goal of maximizing the use of medical treatment and avoiding repeated surgical procedures” (Practice Committee of American Society for Reproductive Medicine, 2008). For this reason it is important to evaluate the efficacy or the long-term safety and tolerability of the medical options available, represented by hormonal therapies. Recent evidence suggest to use an alternative non-hormonal drugs, efficacious as hormonal drugs, but with less systemic side effects.

Current knowledge on the pathophysiology of endometriosis indicates that induction of the disease involves changes in structural elements, a proinflammatory environment, resistance to apoptosis, and increased angiogenesis (Gauché-Cazalis et al., 2012). Palmitoylethanolamide, an endogenous fatty acid amide, has shown in numerous studies significant anti-inflammatory, and analgesic effects in experimental models of chronic and acute neuropathic pain and chronic inflammation (De Filippis et al., 2011), and in treating pathological pain clinically (Skaper et al., 2013). PEA down-regulates mast cell activation and inhibits their degranulation and has recently been reported to reduce viscerovisceral hyperalgesia in a rat model of endometriosis plus ureteral calculus (Iuvone et al., 2016).

However, the PEA does not have antioxidant effects, for this reason it was used a compound that combines the anti-inflammatory action of the PEA to the antioxidant action of 3,4′,5-trihydroxystibene-3-β-mono-D-glycoside (polydatin), a natural glucoside of resveratrol.

It has been demonstrated that PLD protects organs like brain, lung, intestine, and heart against ischemia–reperfusion injury (Miao et al., 2011), and possesses anti-oxidation (Kerem et al., 2006), anti-shock, and anti-inflammatory (Lanzilli et al., 2012) properties. Polydatin also inhibits migration and proliferation of vascular endothelial cells and inhibits the angiogenic process via inhibition of proangiogenic factors such as matrix metalloproteinases (MMPs) and vascular endothelial growth factor (VEGF).

The purpose of this study was to investigate whether oral administration of m(PEA/PLD) could reduce inflammation and pain associated with endometriosis.

## Materials and Methods

### Animals

Sprague–Dawley female rats (200–230 g, Harlan, Nossan, Italy) were used throughout. They received food and water *ad libitum*. The study was authorized by the University of Messina Review Board for the care of animals (Protocol number 8/U-apr16). Animal care followed Italian (D.M.116192) and EEC (O.J. of E.C. L 358/1 12/18/1986) regulations on protection of animals used for experimental and scientific purposes.

### Induction of Experimental Endometriosis

Endometriosis was induced using homologous uterine horn transplantation. All rats were anesthetized with Isoflurane. As described by Rajkumar et al. (1990) laparotomy was performed, the uterus was exposed and the left horn excised. This tissue fragment was placed in phosphate-buffered saline (PBS) 37°C and was cut along the longitudinal axis, obtaining two pieces of 5 × 5 mm. One of the two squares was sutured to the inner surface of the abdominal wall, with the endometrial layer facing a large vessel in the peritoneal cavity, and the other one to the bowel mesentery close to a large vessel. All implants were sutured using a sterile 6-0 silk suture and the abdominal cavity was closed using 4-0 silk suture. Penicillin (40,000 U/kg) was administered for 5 days after the surgery.

### Experimental Groups

Rats were randomized with the technique of “simple randomization” (Ivers et al., 2012) and divided into the following groups (*N* = 10).

#### Control Group

Rats were subjected to experimental endometriosis as described above, and vehicle (carboxymethylcellulose 1.5% w/v in saline) was administered orally by gavage, on the 14 day and for the next 14 days.

#### m(PEA/PLD) Group

Rats were subjected to experimental endometriosis as described above, and m(PEA/PLD) (10 mg/kg) dissolved in carboxymethylcellulose (1.5% w/v in saline) was administered orally by gavage, on the 14 day and for the next 14 days.

The dose of m(PEA/PLD) was based on a previous study (Esposito et al., 2016).

In order to evaluate endometriotic lesions, rats were sacrificed at 28 days after endometriosis induction (Ricci et al., 2013; Ozcan Cenksoy et al., 2015). Rats were anesthetized with isoflurane; laparotomy was performed to collect the endometriotic implants. Implants were excised from both groups, weighed, measured, and processed for histology and biochemical studies.

### Histology

For histopathological investigations, endometriotic implants were collected 28 days post-surgery. Samples were fixed at room temperature in buffered formaldehyde solution (10% in PBS) for 24 h, then dehydrated using a graded series of ethanol, embedded in Paraplast (Sherwood Medical, Mahwah, NJ, USA) and cut into 7-mm thick sections. Sections were deparaffinized with xylene, stained with H&E and analyzed using an Axiovision Zeiss (Milan, Italy) microscope. Damage was estimated by an expert histopathologist blinded to the study, and scored as follows: 0 = no implantation, 1 = cellular infiltration, 2 = edema, 3 = continuous inflammatory lesions, 4 = presence of glandular epithelium, and stroma.

### Masson Trichrome and Toluidine Blue Staining

To evaluate the degree of fibrosis, explant tissue sections were stained with Masson trichrome according to the manufacturer’s protocol (Bio-Optica, Milan, Italy). For evaluation of number and degranulation of mast cells, tissue sections were stained with toluidine blue. Sections were deparaffinized in xylene and dehydrated through a graded series of ethanol, 5 min in each solution. The sections were next placed in water for 5 min, transferred to toluidine blue for 4 min and then blotted carefully. Sections were placed in absolute alcohol for 1 min, cleared in xylene, and mounted on a glass slide using Eukitt (Bio-Optica, Milan, Italy). Sections were stained blue and the mast cells were stained purple. Metachromatically stained mast cells were enumerated by counting five high-power fields (40×) per section using Axiovision Zeiss (Milan, Italy) microscope.

### Immunohistochemical Localization of Nitrotyrosine, poly(ADP-Ribose) (PAR), VEGF, NGF, MMP9, MPO, and ICAM

At 28 days from surgery, endometriotic implants were fixed in 10% (w/v) PBS-buffered formaldehyde and embedded in paraffin. Seven micrometer sections were prepared from tissues. After deparaffinization, endogenous peroxidase was quenched with 0.3% (v/v) hydrogen peroxide in 60% (v/v) water for 30 min. The slides were permeabilized with 0.1% (w/v) Triton X-100 in PBS for 20 min. Tissue sections were incubated in 2% (v/v) normal goat serum in PBS to block non-specific binding. Sequential incubation for 15 min with avidin and biotin (Vector Laboratories, Burlingame, CA, USA) was performed to block, respectively, endogenous avidin, or biotin binding sites. Sections were then incubated overnight with: anti-PAR antibody (H-250: sc-7150, 1: 550 in PBS, Santa Cruz Biotechnology), anti-nitrotyrosine antibody (Millipore, 06-284), anti-VEGF antibody (A-20: sc-152, 1:300 in PBS, Santa Cruz Biotechnology), anti-NGF antibody (E-12: sc-365944, 1:450 in PBS, Santa Cruz Biotechnology), anti-MMP9 antibody (C-20: sc-6840, 1:350 in PBS, Santa Cruz Biotechnology), anti-MPO antibody (2C7: sc-59600, 1:325 in PBS, Santa Cruz Biotechnology), or anti-ICAM antibody (G-5: sc-8439, 1:325 in PBS, Santa Cruz Biotechnology). Samples were washed with PBS, and incubated with secondary antibody. Specific labeling was identified with a biotin-conjugated goat anti-rabbit IgG and avidin–biotin peroxidase complex (Vector Laboratories, Burlingame, CA, USA). Immunohistochemical images were collected using a Zeiss microscope and Axio Vision software. For graphic display of densitometric analyses, the intensity of positive staining (brown staining) was measured by computer-assisted color image analysis (Leica QWin V3, UK). The percentage area of immunoreactivity (determined by the number of positive pixels) was expressed as percent of total tissue area (red staining).

### Western Blot Analysis for Nitrotyrosine, VEGF, NGF, ICAM, IkB-α, and NF-kB

Endometriotic explants were suspended in buffer A (0.2 mM phenylmethylsulfonyl fluoride, 0.15 μM pepstatin A, 20 μM leupeptin, and 1 mM sodium orthovanadate) homogenized for 2 min, and centrifuged at 10000 *g* for 10 min at 4°C. Supernatants represented the cytosolic fraction. The pellets, containing nuclei, were re-suspended in buffer B (150 mM NaCl, 1% Triton X-100, 1 mM EGTA, 1 mM EDTA, 10 mM Tris–HCl pH 7.4, 0.2 mM phenylmethylsulfonyl fluoride, 20 μM leupeptin, and 0.2 mM sodium orthovanadate). After centrifugation for 30 min at 15,000 *g* at 4°C, the supernatants contained nuclear proteins. Samples were stored at -80°C for further analysis. The levels of nitrotyrosine, VEGF, NGF, ICAM, IkB-α, and Bax were quantified in the cytosolic fraction, while the nuclear fraction was used to quantify NF-kB p65 levels. Membranes were blocked with 1× PBS, 5% (w/v) non-fat dried milk for 40 min at room temperature and later probed with one of the following primary antibodies: anti-nitrotyrosine (Millipore, 06-284), anti-VEGF (A-20: sc-152, 1:300 in PBS, Santa Cruz Biotechnology), anti-NGF (Santa Cruz Biotechnology, E-12: sc-365944, 1:450 in PBS, v/v) or anti-ICAM antibody (G-5: sc-8439, 1:325 in PBS, Santa Cruz Biotechnology), anti-Bax (P-19: sc-526, 1:225 in PBS, Santa Cruz Biotechnology), IkB-α (1:500, Santa Cruz Biotechnology) or, anti-NF-kB p65 (1:400; Santa Cruz Biotechnology) in 1× PBS, 5% (w/v) non-fat dried milk, 0.1% Tween-20 at 4°C overnight. Filters were incubated with peroxidase-conjugated bovine anti-mouse IgG secondary antibody or peroxidase-conjugated goat anti-rabbit IgG (1:5000, Jackson ImmunoResearch, West Grove, PA, USA) for 1 h at room temperature. To establish that blots contained equal amounts of protein they were also incubated with an antibody against β-actin (1:500, Sigma–Aldrich). Relative expression of protein bands for nitrotyrosine (67 kDa), VEGF (34 kDa), NGF (13 kDa), ICAM (90 kDa), IkB-α (37 kDa), NF-kB p65 (65 kDa), and Bax (23 kDa) was quantified by densitometric scanning of the X-ray films with GS-700 Imaging Densitometer (GS-700, Bio-Rad Laboratories, Milan, Italy) and a Image Lab 3.0 software (Bio-Rad, USA), and standardized to β-actin levels.

### Quantification of Pain Behaviors

#### Uterine Pain Behaviors

Uterine pain behaviors were evaluated looking at four positions: “lambda” position (the rat suddenly hunches its back upward into a sharp angle to forma triangular shape relative to the floor), “alpha” position (rat with abdomen adherent to the floor and nose curving toward the tail of the affected side), “stretch-flat” position, with stretching of the body with abdomen adherent to the floor, and “squash-pelvic” position (squashing of the lower part of the abdomen to the floor while in a standing or sitting position). For each rat, relative to the whole period of recording, the uterine pain behaviors are characterized in terms of global duration of uterine positions (sum of duration of all positions; Giamberardino et al., 2002).

#### The Tail-Fick Method

The warm water tail-flick test was used to determine pain threshold. Test was conducted before and during treatment. 4 cm of the rat-tail was placed in 50 ± 0.5°C warm water and the time between tail input and withdrawal from the water was recorded (three tests were conducted and the average in units of seconds was recorded). The latency was recorded with a sensitivity of 0.01 s. To perform a measurement, the rat had to remain calm, without unconditional unexpected movements of the tail. A maximum tail-flick latency of 10 s was used to minimize tissue damage to the tail (Liu et al., 2012).

#### The Hot-Plate Method

The hot-plate latency was assessed by placing the rat on a metal surface maintained at 53.6°C (Hot Plate, Ugo Basile, Milan, Italy). The rat was observed closely during the measurement period and the licking of a hind paw was taken as the end point. At this moment the latency to respond was recorded and the rat was immediately removed. Maximal latency accepted was 45 s (Liu et al., 2012).

### Materials

Co-micronized Palmitoylethanolamide/Polydatin was obtained from Epitech Group SpA (Saccolongo, Italy). All compounds were obtained from Sigma-Aldrich (Milan, Italy). All chemicals were of the highest commercial grade available. All stock solutions were prepared in non-pyrogenic saline (0.9% NaCl; Baxter, Italy, UK).

#### Statistical Evaluation

All values are expressed as mean ± standard error of the mean (SEM) of *N* observations. For *in vivo* studies *N* represents the number of animals used. For histological experiments, the figures shown are representative of at least three experiments (histological staining) performed on different days on tissue sections collected from all animals in each group. The results were analyzed by 2-way ANOVA. A *P*-value of less than 0.05 was considered significant.

## Results

### Effects of m(PEA/PLD) Treatment on the Degree of Endometriosis

**FIGURE 1:**
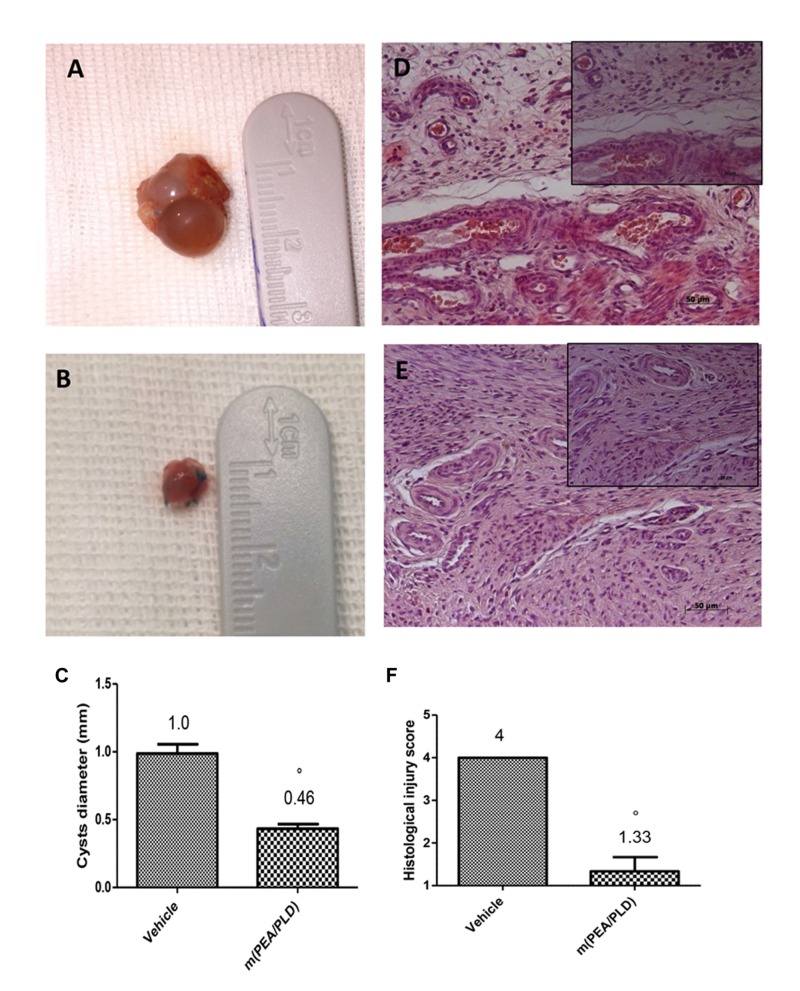
**Effect of m(PEA/PLD) treatment on cyst diameter and histological damage score.** m(PEA/PLD) treatment significantly reduced cyst size (0.46 mm) **(B,C)** when compared with vehicle-treated control rats (1.0 mm) **(A,C)** (°*p* = 0.01). Histological examination showed the presence of endometrial tissue containing glandular epithelium and stroma. Cysts of the control group contained cellular infiltration and edema (4) **(D,F)**, and m(PEA/PLD) reduced the severity of histological marks of endometriotic cysts (1.33) **(E,F)** (°*p* = 0.01). Data are means ± SD of 10 rats for each group. °*P* < 0.05 vs. vehicle group.

At 28 days post-surgery all uterine implants displayed transparent cystic areas. Both control (**Figure 1A**) and m(PEA/PLD) (**Figure 1B**) groups developed cysts in the mesentery and abdominal wall. The two groups did not differ in cysts number, although cyst size was smaller in m(PEA/PLD)-treated rats compared to control rats (*P* < 0.05; **Figure 1C**). Histological examination showed the presence of endometrial tissue containing glandular epithelium and stroma. Cysts in the control group were characterized by an intense cellular infiltration, edema and continuous inflammatory lesions (**Figure 1D**, see histological injury score in panel 1F). m(PEA/PLD) treatment reduced the level and severity of the macroscopic and histological marks of endometriotic cysts (**Figure 1E**, see histological injury score in **Figure 1F**).

### Effects of m(PEA/PLD) Treatment on Fibrosis and MMP9 Expression

**FIGURE 2:**
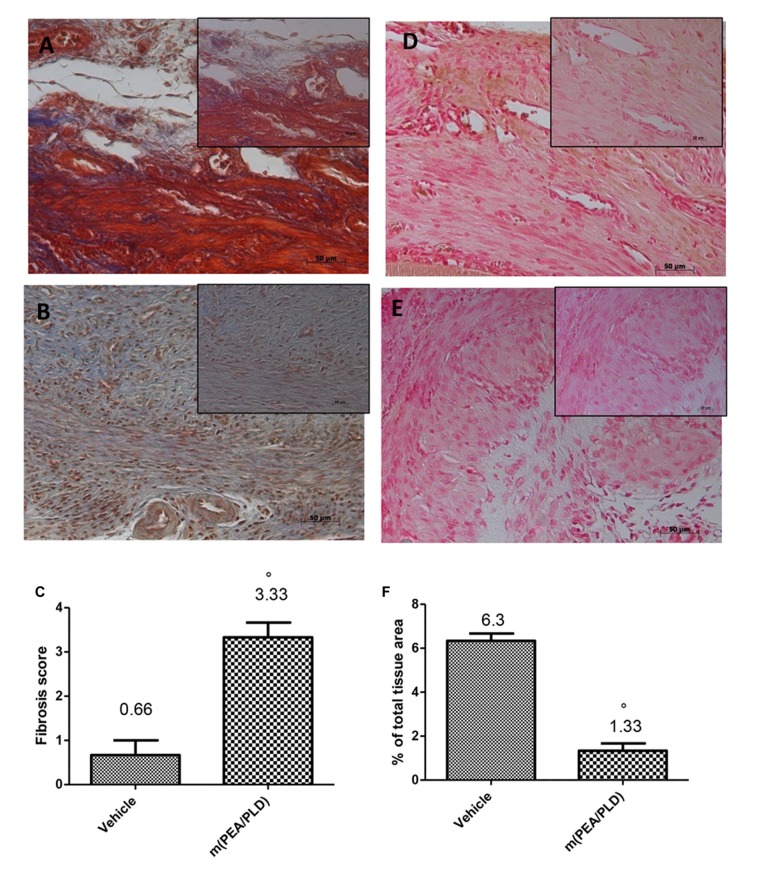
**Effect of m(PEA/PLD) treatment on Masson trichrome and MMP9 expression.** Masson trichrome staining showed that tissues from the endometriotic control group displayed fibrosis (0.66) **(A,C)**, which was more pronounced in the m(PEA/PLD) group (3.33) (°*p* = 0.01) **(B,C)**. Positive staining for MMP9 was significantly increased in vehicle-treated rats (6.3) **(D,F)**, whereas m(PEA/PLD) (10 mg/kg) reduced the degree of positive staining for MMP9 in cysts (1.33) **(E,F)** (°*p* = 0.01). Data are means ± SD of 10 rats for each group. °*P* < 0.05 vs. vehicle group.

In endometriotic lesions 28 days post-implantation the degree of fibrosis, assessed by Masson trichrome staining (**Figures 2A,B**) demonstrated a fibrotic area stained blue that was larger in the m(PEA/PLD) group (**Figures 2B,C**) than in the control group. Moreover, immunohistochemical analysis showed a significantly increased MMP9 expression (a marker of inflammation, especially in infiltration of inflamed tissues) in endometriotic tissues from control rats (**Figures 2D,F**), whereas m(PEA/PLD; 10 mg/kg) reduced the degree of positive staining for MMP9 (**Figures 2E,F**).

### Effects of m(PEA/PLD) Treatment on Mast Cells Fensity and NGF Expression

**FIGURE 3:**
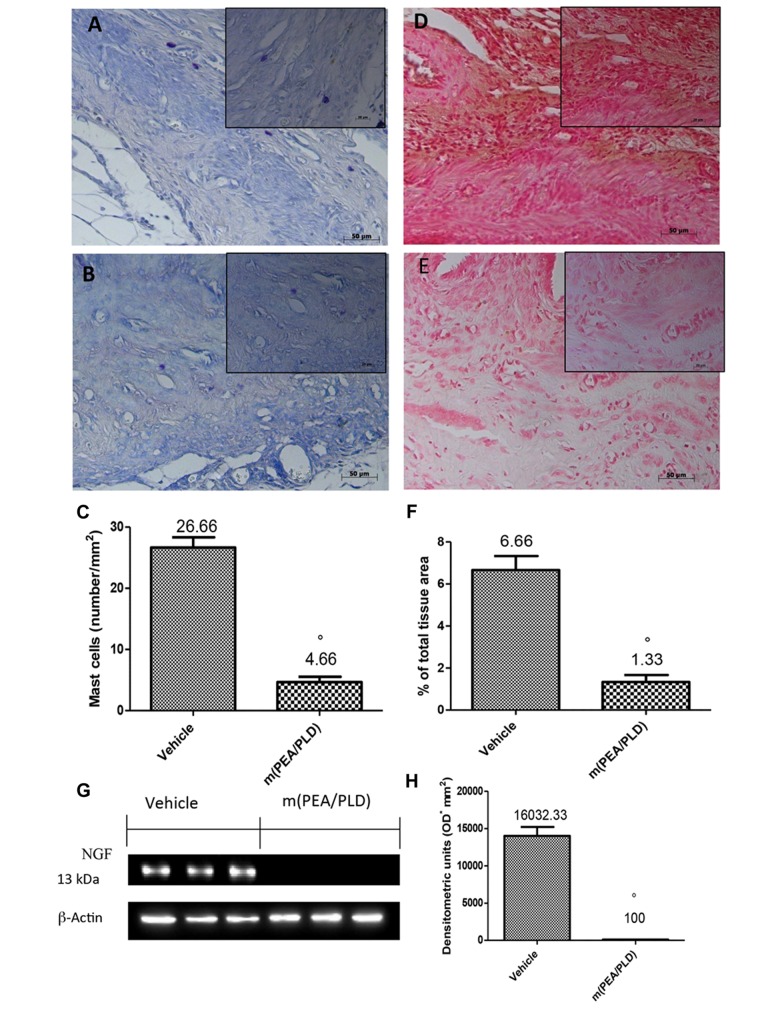
**Effect of m(PEA/PLD) treatment on mast cells and NGF expression.** Toluidine blue staining revealed that mast cell numbers in the endometriotic tissues from the m(PEA/PLD) group (4.66) **(B,C)** was significantly reduced compared to the control group (26.66) **(A,C)** (°*p* = 0.01). NGF staining was significantly increased in vehicle-treated rats (6.66) **(D,F)**, whereas m(PEA/PLD) (10 mg/kg) reduced the extent of staining for NGF (1.33) **(E,F)** (°*p* = 0.01). Western blot analysis showed that NGF levels were considerably higher in samples from vehicle-treated control rats (16032.33), while m(PEA/PLD) treatment decreased NGF expression (100) **(G,H)** (°*p* = 0.01). Data are means ± SD of 10 rats for each group. °*p* < 0.05 vs. vehicle group.

Mast cells numbers in endometriotic tissues were evaluated by staining with toluidine blue (**Figures 3A,C**). Compared with the control group, the number of mast cells in the m(PEA/PLD) group was significantly reduced (**Figures 3B,C**). There is evidence to suggest that NGF can trigger mast cell degranulation (Lin et al., 2015). Immunostaining for NGF was significantly increased in endometriotic tissues from control rats (**Figures 3D,F**), whereas m(PEA/PLD; 10 mg/kg) reduced such positive staining (**Figures 3E,F**). NGF expression in endometriotic explants from control rats was also increased by Western blot analysis, and was reduced by m(PEA/PLD) treatment (**Figures 3G,H**).

### Effects of m(PEA/PLD) Treatment on VEGF, ICAM, and MPO Expression

**FIGURE 4:**
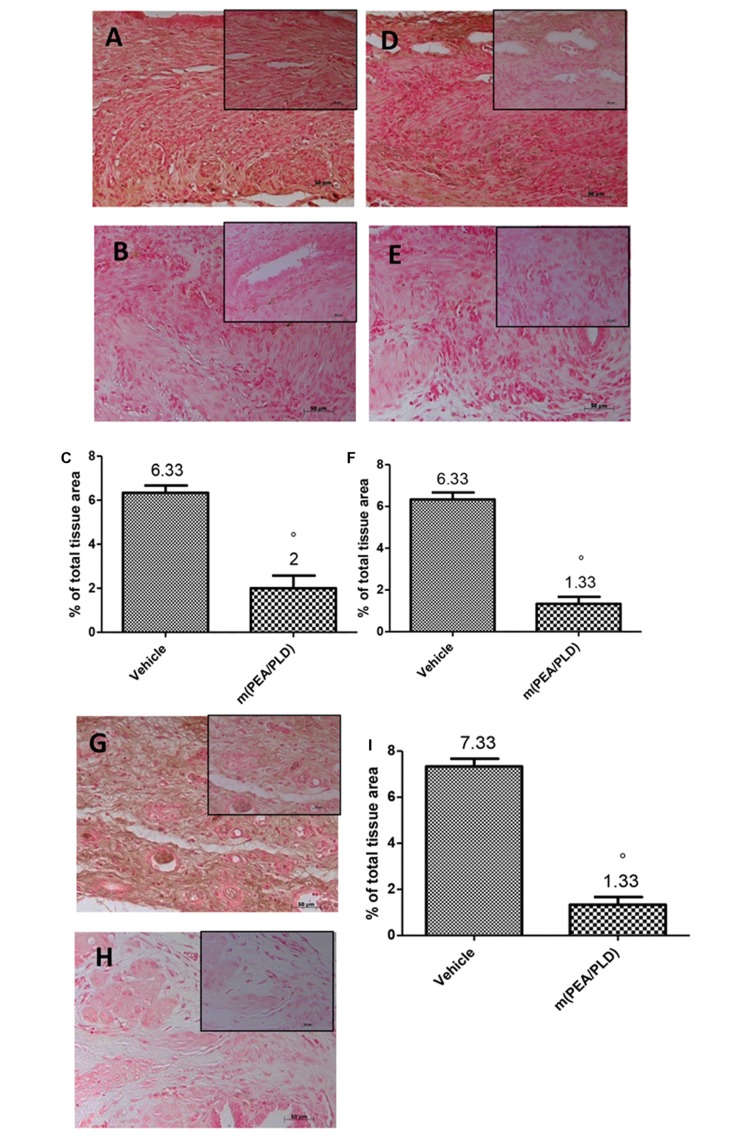
**Effect of m(PEA/PLD) treatment on VEGF, ICAM and MPO expression.** Positive staining for VEGF was considerably increased in cyst tissue from vehicle-treated control rats (6.33) **(A,C)**, m(PEA/PLD) (10 mg/kg) reduced this positive staining (2) **(B,C)** (°*p* = 0.01). Positive staining for ICAM-1 was higher in tissues collected from control rats (6.33) **(D,F)**, and treatment with m(PEA/PLD) reduced this positive staining for ICAM-I (1.33) **(E,F)** (°*p* = 0.01). Positive staining for MPO was markedly increased in cyst tissues from vehicle-treated control rats (7.33) **(G,I)**; m(PEA/PLD) significantly reduced polymorphonuclear cell infiltration as MPO positive staining (1.33) **(H,I)** (°*p* = 0.01). Data are means ± SD of 10 rats for each group. °*p* < 0.05 vs. vehicle group.

Immunohistochemical analysis of the endometriotic lesions 28 days after implantation demonstrated increased staining for the main proangiogenic factor VEGF in endometriotic tissues from control rats (**Figures 4A,C**), which was reduced by m(PEA/PLD) (10 mg/kg) treatment (**Figures 4B,C**). To better clarify the effect of m(PEA/PLD) on endothelial cells ICAM-1 expression was examined. Positive immunostaining for ICAM-1 was significantly increased in the vessels of sub mucosa and lamina propria in tissues collected from control rats (**Figures 4D,F**). Treatment with m(PEA/PLD) (10 mg/kg) reduced ICAM-1 staining (**Figures 4E,F**). Endometriotic lesions exhibited an elevated accumulation of neutrophils, as assessed by MPO activity, a reliable indicator of polymorphonuclear cell infiltration in endometriotic lesions. A marked staining for MPO was observed in endometriotic tissues from control rats (**Figures 4G,I**), which was significantly reduced m(PEA/PLD) (10 mg/kg) (**Figures 4H,I**).

### Effects of m(PEA/PLD) Treatment on IκB-*α* Degradation and NF-κB Activation

**FIGURE 5:**
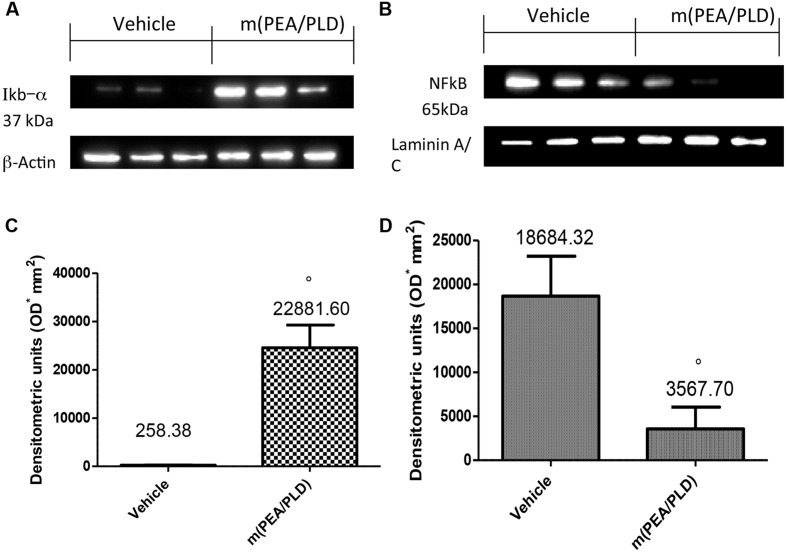
**Effect of m(PEA/PLD) treatment on IκB-*α* degradation and NF-κB.** Cyst samples from vehicle-treated control rats displayed low IκB-α expression (2591.71) while m(PEA/PLD) treatment did not allow IκB-α degradation (22881.60) **(A,C)** (°*p* = 0.03). NF-κB levels in endometriotic cyst nuclear fractions (18684.32) were noticeably increased 28 days after implantation; m(PEA/PLD) treatment significantly decreased NF-κB levels (3567.70) **(B,D)** (°*p* = 0.02). Data are means ± SD of 10 rats for each group. °*p* < 0.05 vs. vehicle group.

To investigate the cellular mechanisms by which m(PEA/PLD) treatment may attenuate the development of endometriosis, we analyzed IκB-α degradation and NF-κB expression by Western blot analysis. Cyst samples from control-rats showed only low IκB-α expression compared to the m(PEA/PLD)-treated group where IκB-α was not (**Figures 5A,C**). NF-κB levels in endometriotic tissues were noticeably increased 28 days after implantation. m(PEA/PLD) treatment significantly decreased NF-κB levels (**Figures 5B,D**).

### Effects of m(PEA/PLD) Treatment on Nitrotyrosine and PAR Formation

**FIGURE 6:**
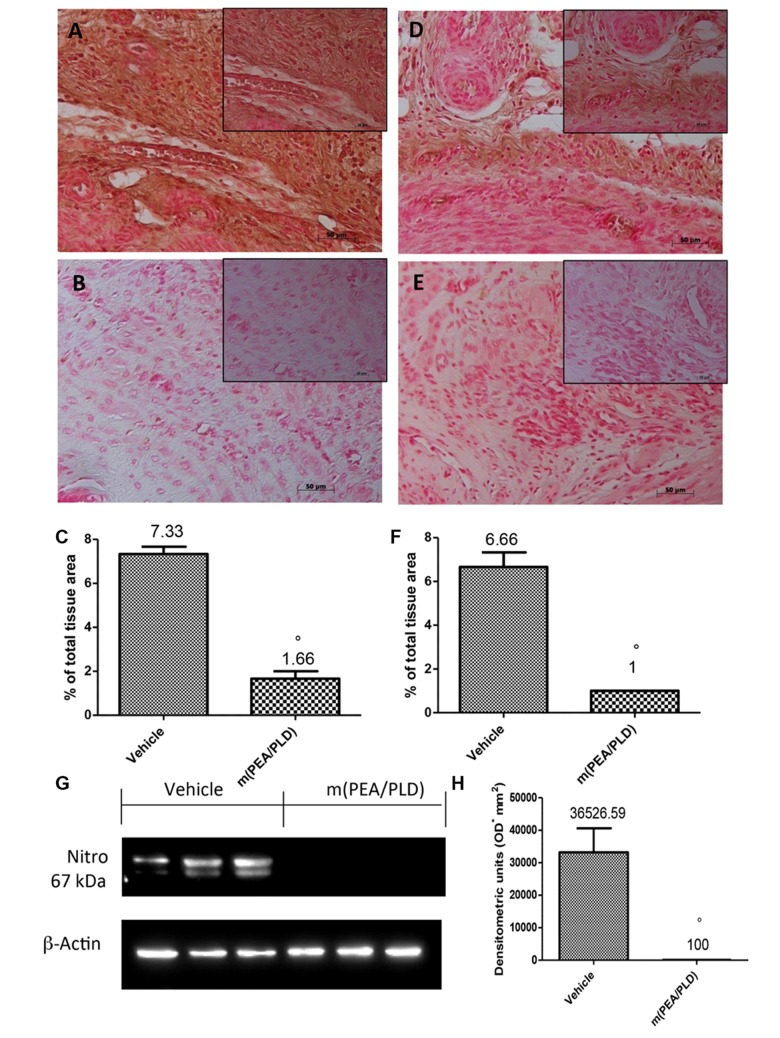
**Effect of m(PEA/PLD) treatment on nitrotyrosine and PAR formation.** Cyst sections from vehicle-treated control rats revealed positive staining for nitrotyrosine (7.33) **(A,C)**, whereas m(PEA/PLD) (10 mg/kg) reduced this staining (1.66) **(B,C)** (°*p* = 0.01). Western blot analysis **(G,H)** revealed a significant elevation in nitrotyrosine expression in vehicle-treated control rats (36526.59) while m(PEA/PLD) reduced its expression (100) (°*p* = 0.04). Positive staining for PAR was clearly increased in nuclei of inflammatory cells in cyst tissues from vehicle-treated control rats (6.66) **(D,F)**, while m(PEA/PLD) reduced such staining (1) **(E,F)** (°*p* = 0.01). Data are means ± SD of 10 rats for each group. °*p* < 0.05 vs. vehicle group.

To define the presence of nitrogen derivatives and/or “peroxynitrite formation” in the endometriotic lesions 28 days after implantation, immunohistochemical analysis was applied to identify nitrotyrosine, a specific marker of nitrosative stress. Endometriotic explants from control rats revealed positive nitrotyrosine staining (**Figures 6A,C**), whereas m(PEA/PLD) (10 mg/kg) treatment resulted in a significant reduction (**Figures 6B,C**). Further, Western blot analysis revealed a significant increase in nitrotyrosine expression (**Figures 6G,H**) in control rats, which was reduced in the m(PEA/PLD) group. To investigate poly(ADP-ribose)polymerase (PARP) activation PAR formation was analyzed immunohistochemically. PAR staining was significantly increased in the nuclei of inflammatory cells in cyst tissues collected from control rats (**Figures 6D,F**), and m(PEA/PLD) (10 mg/kg) treatment reduced this rise in PAR staining (**Figures 6E,F**).

### Effect of m(PEA/PLD) Treatment on Pain Behaviors

**FIGURE 7:**
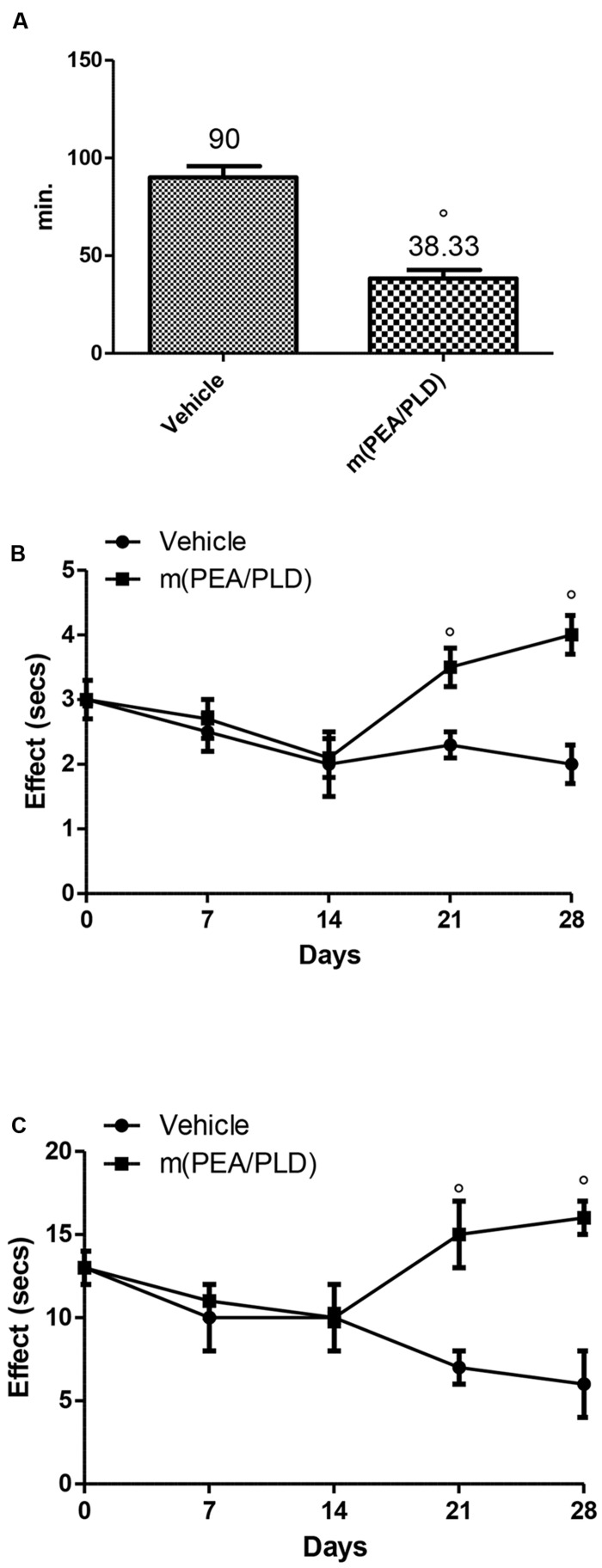
**Effect of m(PEA/PLD) treatment on pain behaviors.** Animals treated with m(PEA/PLD) displayed less pain behaviors than vehicle-treated control rats. The baseline nociception and the antinociceptive effect after m(PEA/PLD) administration were measured looking at the uterine pain behaviors **(A)**, with the tail-flick method **(B)** and the hot plate method **(C)**. Vehicle-treated control rats were affected by longer uterine crises than m(PEA/PLD) treated rats **(A)** (°*p* = 0.010). During the tail-flick **(B)** and hot plate **(C)** tests animals administrated with m(PEA/PLD) showed a significant antinociceptive effect compared to the vehicle-treated control rats (°*p* = 0.004). Data are means ± SD of 10 rats for each group. °*p* < 0.05 vs. vehicle group.

To investigate directly the reduction of pain produced by the m(PEA/PLD) treatment immediately after implantation we analyzed the animals’ behaviors. The m(PEA/PLD) treated rats showed significantly lower duration of uterine pain behaviors than vehicle-treated control animals (**Figure 7A**). m(PEA/PLD) resulted antinociceptive effect in tail-flick (**Figure 7B**) and hot plate test (**Figure 7C**) already after 7 days of treatment (21 days after implantation), which increased at 14 days of treatment (28 days after implantation). Administration of vehicle had no effect on nociceptive threshold.

## Discussion

Implantation, proliferation, angiogenesis, and inflammation play a central role in the initiation and growth of endometriotic lesions (Delbandi et al., 2013). Using an autologous rat model of surgically induced endometriosis, we show that m(PEA/PLD) treatment brought about a significant decrease in cyst diameter and reduced histopathological score. In this model the degree of fibrosis is another important pathological index (Zheng et al., 2016), and m(PEA/PLD) treatment improved also fibrosis score in endometriotic lesions. The adherence and invasion of endometriotic cells require an over-production of MMPs. MMPs, a family of endoproteases, have been suggested to be involved in basement membrane and extracellular matrix degradation, which are crucial steps for cellular invasion and migration. The expression of several MMPs, especially MMP9, has been demonstrated in endometriosis (Wu et al., 2005). Our results confirmed MMP9 up-regulation and showed that treatment with m(PEA/PLD) significantly reduced immunostaining of MMP9. Mast cells appear to play an important role in the development of endometriosis (Howard, 2009) and have been found in the implanted tissue, proximal to nerves, and are thus well placed to release numerous mediators, such as NGF, and thereby contribute to endometriotic pelvic pain (Bokor et al., 2009). m(PEA/PLD) administration decreased mast cell number in endometriotic cysts, compared to vehicle-treated rats. This finding is in line with reported analgesic effects of PEA. Indeed, numerous studies have shown that PEA by the modulation of mast cells activation controls pain perception (Bettoni et al., 2013). Treatment with m(PEA/PLD) led to, a significant reduction of NGF expression in endometriotic cysts paralleled the decrease in mast cell numbers.

Mast cells play a central role in neoangiogenesis, another endometriosis feature, which guarantees oxygen supply to lesions (Groothuis et al., 2005). Histological analysis of endometriotic cysts from rats receiving m(PEA/PLD) demonstrated a strong antiangiogenic effect. Conceivably, VEGF down-regulation was due to mast cell modulation by m(PEA/PLD). VEGF is released by mast cells during chronic inflammation (Dmowski and Braun, 2004), and this reduced angiogenesis may be responsible for the reduction in cyst diameter in m(PEA/PLD)-treated animals. New vessel formation is required for delivery of nutrients and oxygen to cysts, facilitating their implantation and development. Increasing evidence points to a crucial role for ICAM-1 in the pathological processes underlying endometriosis (Mousa, 2003). Because treatment with m(PEA/PLD) reduced ICAM-1 up-regulation, m(PEA/PLD) could reduce the interaction between neutrophils and endothelial cells mediated by ICAM-1 at the adhesion phase. These results are consistent with the reduced leukocyte infiltration in endometriotic cysts indicated by MPO, an important enzyme used by granulocytes during phagocytic lysis of engulfed foreign particles. In endometriotic lesions both neutrophilic and eosionophilic myeloid cell types, at all stages of maturation, exhibit strong cytoplasmic reactivity for MPO (Berkes et al., 2014). m(PEA/PLD) treatment decreased MPO reactivity in endometriotic cysts, compared to vehicle-treated rats. The protective effects of m(PEA/PLD) may be attributable, in part, to suppression of the inflammatory response via down-regulation of NF-kB (Esposito and Cuzzocrea, 2013). Our results confirmed that NF-kB is activated in endometriotic cysts of rats, an effect significantly reduced by m(PEA/PLD) administration. m(PEA/PLD), by reducing NFkB translocation in the nucleus, decreased the phosphorylation of IkBα. This result indicated that m(PEA/PLD) is able to control oxidant/antioxidant balance.

Endometriotic lesions involve an over-production of ROS, which cause oxidation of cellular components such as proteins, lipids, and DNA, resulting in cell damage. The most common ROS are superoxide anion and peroxynitrite. Cellular targets of peroxynitrite include carbohydrates, proteins, lipids, and nucleic acids, whose reaction of peroxynitrite results in protein modification by oxidization of sulfhydryl groups, lipid peroxidation and nitration of tyrosine residues. Lipid peroxidation and nitrotyrosine formation were elevated in endometriotic tissues, and treatment with m(PEA/PLD) significantly reduced immunostaining of nitrotyrosine. Peroxynitrite can activate PARP, a DNA repair enzyme that responds to single-strand breaks in DNA by synthesizing chains of ADP-ribose that serve as an indicator for other enzymes of DNA repair. As NAD+ is an obligate substrate to in the production of ADP-ribose monomers, hyper-activation of PARP can deplete cellular reserves of NAD+ and lead to ATP depletion. Depletion of ATP and NAD caused by this activation leads to cellular dysfunction and death. In the present study, m(PEA/PLD) decreased PARP activity in endometriotic cysts. One needs to keep in mind that apoptosis plays a key role in the endometriotic process (Wang et al., 2016).

It has been demonstrated that the PEA–PLD combination seems to be very useful in controlling chronic pelvic pain associated with endometriosis (Giamberardino et al., 2010; Indraccolo and Barbieri, 2010). In agreement with previous results, in our study we showed that m(PEA/PLD) treatment, compared with vehicle, significantly reduced the behavioral indicated of uterine pain and animals showing no signs of chronic suffering.

Palmitoylethanolamide has been explored in man in various clinical trials in a variety of pain states, for inflammatory and pain syndromes (Indraccolo and Barbieri, 2010). PEA is available for human use as a supplement and as food for medical purposes.

Our data clearly demonstrate that treatment with m(PEA/PLD) reduced the inflammatory process and pain associated with an experimental rat model of surgically induced endometriosis. Thus we propose that the m(PEA/PLD) as witnessed for the assessment of pain, can enter into a clinical study to verify the effectiveness of this treatment in controlling the inflammatory process associated with endometriosis in woman.

## Author Contributions

RD gave substantial contributions to the conception and design of the work and approved the version to be published. RF and EG performed the experiments and approved the version to be published. ME and RC analyzed data and approved the version to be published. RG drafted the work and approved the version to be published. SC revised it critically for important intellectual content and approved the version to be published.

## Conflict of Interest Statement

SC is co-inventor on patent WO2013121449 A8 (Epitech Group Srl) which deals with methods and compositions for the modulation of amidases capable of hydrolysing *N*-acylethanolamines employable in the treatment of inflammatory diseases. This invention is wholly unrelated to the present study. Moreover, SC is also, with Epitech Group, a co-inventor on the following patent: EP 2 821 083; MI2014 A001495; 102015000067344 that are, however, unrelated to the study. All the authors declare that the research was conducted in the absence of any commercial or financial relationships that could be construed as a potential conflict of interest.

The reviewer LM declared a shared affiliation, though no other collaboration, with the authors to the handling Editor, who ensured that the process nevertheless met the standards of a fair and objective review.

## Abbreviations

H&E: Hematoxylin/eosin
HCl: hydrochloric acid
ICAM: intracellular adhesion molecule
m(PEA/PLD): co-micronized PEA/polydatin
MPO: myeloperoxidase
NaCl: sodium chloride
NGF: nerve growth factor
PAR: (poly-ADP)ribose polymerase activation
PEA: palmitoylethanolamide
PLD: polydatin
ROS: reactive oxygen species
